# Security exchange commission forms K-10 filings – Positive and negative word occurrence dataset 1995–2008

**DOI:** 10.1016/j.dib.2022.108110

**Published:** 2022-03-29

**Authors:** Piotr Staszkiewicz, Richard Staszkiewicz

**Affiliations:** a Collegium of Business Administration, Institute of Corporate Finance and Investment, SGH Warsaw School of Economics, Poland; b The Faculty of Electronics and Information Technology, Warsaw University of Technology, Poland

**Keywords:** Ton, Sentiment, Form 10-K, Text mining, Positive list, Negative list, US GAAP, EDGAR

## Abstract

Corporate disclosure became more descriptive rather than quantitative over time. Thus, textual analysis gained popularity in finance and business, however, it requires massive computing power. The paper presents the panel set of the raw frequencies of positive and negative words across 90,463 Forms 10-K filed at Security Exchange Commission (SEC) in EDGAR (the Electronic Data Gathering, Analysis, and Retrieval system) over the period 1995–2008. The dataset consists of 456 variables. The texts of the forms were retrieved from the SEC servers and processed using text mining techniques. The data relevant for archive analysis on the sentiment of the financial statements and financial reporting on SEC registrants. Potential reuse for creation of the tone or sentiments indexes. Long-time data series allows for dynamic analysis. The data set allows reducing the computer power requirements for further research.

## Specifications Table

**Table:** 

Subject	*Finance and Banking*
Specific subject area	*Corporate reporting sentiment. Words frequencies and text mining*
Type of data	Table
How the data were acquired	*10-K filings to Security Exchange Commission Electronic Data Gathering, Analysis (EDGAR) system. Fillings were retrieved and analysed with application of the R programming language “edgar” package*
Data format	*Analysed*
Description of data collection	*All available 10-K forms* over the period 1995-2008 were retrieved from EDGAR and analysed. *212 positive entries out of 2005 and 241 negative entries out of 2329, so 10% of the most frequent words from both dictionaries reported in the dataset.*
Data source location	University Campus, Warsaw School of Economics, Poland
Data accessibility	*Repository name: Data for: Security Exchange Commission Forms K-10 filings - dataset of positive and negative words occurrence 1995-2008* *Data identification number: 10.17632/kb9ss2nn6j.1* *Direct URL to data:*https://data.mendeley.com/datasets/kb9ss2nn6j/1 *Raw data available form the EDGAR based on the combined index of CIK, date, type of form: 10-K.*
Related research article^(^a)[2]	P. Staszkiewicz, R. Karkowska, Audit fee and banks’ communication sentiment, Econ. Res. Istraživanja. (2021), on-line first, 10.1080/1331677X.2021.1985567

(a) A subset of data used in the paper.

## Value of the Data

• Dataset is potentially reusable for the sentiment, tone archive research on the US market before the financial crisis.
• Researchers, analysts, and non-profit organisations with computational limitations can benefit from the data, as its use does not require significant computing power.
• The raw frequency allows different construction mechanics of the tone or sentiment indexes.
• Potential usage for machine learning and artificial intelligence systems for an investment decision. Data is likely to be suitable for linguistic studies on technical language development.
• A subsample of the dataset is usable for the robustness test of the sentiment research.

## 1 Data Description

The textual analysis requires a substantial amount of text data and computer power. This dataset reduces those requirements by providing raw data of quantities per each SEC Form 10-K filing from the period 1995–2008. We retrieved the original filing text files with “edgar” R [2] package version 1.0.8 [3]. Excel output was computed for each individual K-10 Form filing positive and negative word frequencies.

The Excel data file consists of the following sheets: (a) corpus, (b) positive and negative words lists identified in Forms 10-K, (c) positive list dictionary used, (d) negative list dictionary used, and (e) variables definitions.

Corpus consists of 456 variables out of which 451 variable relates to the negative and positive words list. There are four technical variables:

File_name – represents a path to the source text of the Form 10-K. The file name consists of CIK_10-K_dateOffiling.

Year – represents the filing year (four digits).

Date – represent the date the Form 10-K was filed in format DD/MM/YYYY.

Intentional_break_end_of_positive – empty variable represents the split in the dataset between positive and negative words.

## 2 Experimental Design, Materials, and Methods

We perform all Forms 10-K analysis filed in the years 1995 to 2008. We retrieved the original filing text files with EDGAR R [3] package version 1.0.8 [4]. The filing text files were coded with the CIK number and date of the Forms 10-K's filing. We applied both a positive and negative dictionary as suggested by Loughran and McDonald Financial Sentiment Dictionaries^1^ and as implemented in the R EDGAR package [5], [6], [7]. We imposed a frequency limit for both dictionaries to avoid biases originating from an individual firm communication style and specific jargon. The identified positive lists comprise 212 entries, while the negative 241 entries represent the 10% tier top of the full lists 2,005 and 2,329 entries respective. The computation was performed on virtual machines with the application of Statistica [8].

**Fig. 1:**
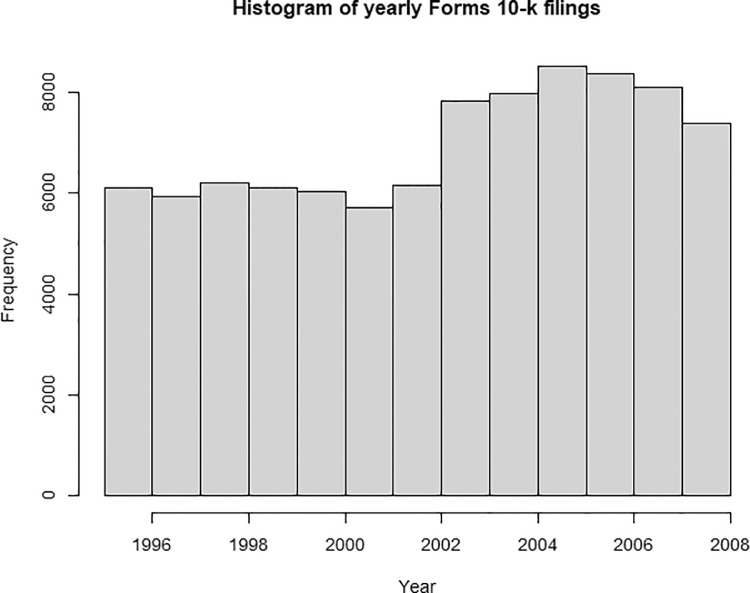
Annual frequency of Forms 10-K filings.

The source text download from EDGAR was randomly checked on the consistency with the CIK and date values against the source documents. The potential inconclusive words in positive and negative lists were removed. Data clean up with the following potentially double-meaning words: *correct* – positive meaning “true” or negative meaning “amendment”; *fine* – positive mining “tiny”, “correct”, negative meaning “penalty”; *restructure* – positive “improvement”, negative “deficiency”. The raw frequency was presented without any data imputation procedure. Fig. 1 presents the histogram of Form 10-K by filing years.

**Fig. 2:**
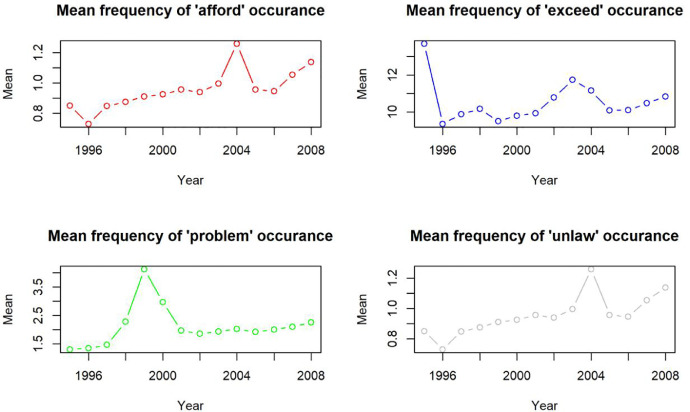
Mena frequency for “afford”, “exceed”, “problem” and “unlaw”.

Fig. 2 shows examples of the mean frequencies by year for positive words: “afford”, “exceed” and negative words: “problem” and “unlaw”.

The figures as stated are one of many uncovering thought-provoking characteristics. Data mining dataset potential is huge, enabling researchers in fields covering not only sentiment analysis and investment decision, but also linguistic studies on technical language development.

## CRediT authorship contribution statement

**Piotr Staszkiewicz:** Conceptualization, Software, Writing – original draft, Writing – review & editing, Validation. **Richard Staszkiewicz:** Software, Formal analysis, Data curation, Writing – original draft, Validation.

## Declaration of Competing Interest

The authors declare that they have no known competing financial interests or personal relationships that could have appeared to influence the work reported in this paper.

## Data Availability

Data for: Security Exchange Commission Forms K-10 filings - dataset of positive and negative words occurrence 1995-2008 [1] (Original data) (Mendeley Data).

## Footnotes

1 https://sraf.nd.edu/textual-analysis/
